# Bone lymphoma revealed by cruralgia during pregnancy: a case report

**DOI:** 10.11604/pamj.2014.18.205.2294

**Published:** 2014-07-06

**Authors:** Ghita Sqalli Houssaini, Latifa Tahiri, Abdelhalim Ibrahimi, Siham Tizniti, Abdelmajid Elmrini, Taoufik Harzy

**Affiliations:** 1 Rheumatology department, CHU Hassan II, Fez, Morocco; 2 Orthopedics surgery department B4, CHU Hassan II, Fez, Morocco; 3 Radiology department, CHU Hassan II, Fez, Morocco

**Keywords:** Bone lymphoma, pregnancy, Non Hodgkin lymphoma

## Abstract

Pregnancy complicated by Non Hodgkin lymphoma (NHL) is rare, about 100 cases have been reported. We will describe the case of a multifocal bone lymphoma revealed by a left hyperalgetic and deficient cruralgia in a female in the second trimester of pregnancy.

## Introduction

Diagnosis of cancer during pregnancy is a relatively rare phenomenon with an incidence of approximately 1 in 1000-1500 pregnancies [1]. It is the second most common cause of maternal death after gestation-related vascular complications, and puts at risk the vital prognosis of the mother and the fetus. The NHL is quite exceptional during pregnancy as well as bone involvment. We will describe a case of a 40-year-old woman with multifocal bone lymphoma who was diagnosed at the 21dt week of gestation revelated by hyperalgetic and deficient cruralgia.

## Patient and observation

**Figure 1:**
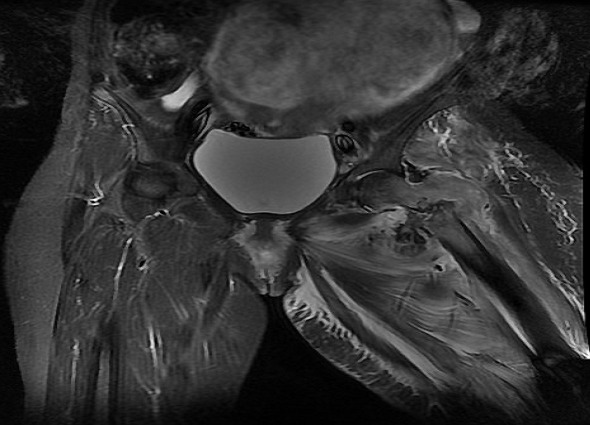
MRI of the thigh, coronal T2 showing a tissular process centred in the left trochanteric region in hypersignal associated with a pathological fracture

**Figure 2:**
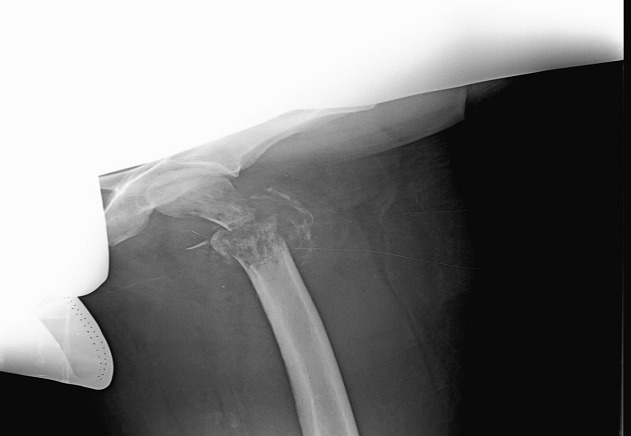
Standard Radiography of the left thigh from the front indicates an osteolytic image at the level of the metaphyseal region of the femur complicated by a displaced pathological pertrochanteric fracture

**Figure 3:**
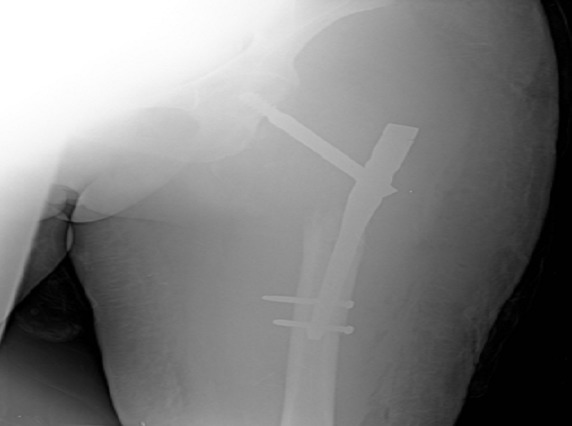
Radiography of the left thigh from the front after an osteosynthesis with a gamma nail

A 40 year old woman, multigravida, is hospitalised at the 21^st^ week of amenorrhoea because of a left cruralgia characterised by electric shocks progressing thirty days before her admission and progressively increasing in intensity, becoming permanent and disabling after twenty days. All progressing in a context of apyrexia and conservation of the overall condition. The clinical examination has revealed a limited abduction external rotation in the left lower limb and left crural deficiency. The biological check-up has shown an inflammatory anaemia, a CRP (C Reactive Protein) at 185mg/l and an erythhrocyt sedimentation rate at 90mm 1h. The pelvic-spine Magnetic resonance imaging (MRI) has demonstrated a tumoral process of the left trochanteric region complicated by pathological fracture (Figure 1) associated with lymph nodes of the common femoral artery and iliac external homolateral artery and focal lesions of the spongious of the right femoral head, the bodies of L1, T10 and T12. A CT-scan guided biopsy of the tumoral mass has shown a diffused Large B cell lymphoma. Subsequent to a multidisciplinary decision between rheumatologists, orthopedic surgeons, gynecologists and hematologists, a therapeutic interruption of pregnancy was realized at the 24^th^ week of amenorrhea followed by tubal ligation according to the wishes of the patient. The fetus (weight 850g) was dead one hour after the birth due to a respiratory distress. An osteosynthesis with a long gamma nail for hip fracture was realized (Figure 2 and Figure 3) then she was transferred to hematology department. Afterwards a cervico-thoraco-abdomino-pelvic scanner which has not shown any other visceral or lymph nodes involvement, a R-CHOP (Rituximab, cyclophosphamide, doxorubicin, vincristine, prednisolone) chemotherapy was started.

## Discussion

Cruralgia during pregnancy is very rare. If the patient presents a neurological deficit or a disturbance of the biological check-up entail, a pelvic ultrasonography and a pelvic-spine magnetic resonance imaging (MRI) should be realized, in order to objective a compression of the femoral nerve at its origin or in the course of its path by an infectious or tumoral process [2].

Our patient was diagnosed with a Non Hodgkin's lymphoma. This haematological malignancy is very rare during pregnancy. It comes in the fourth position after cervical cancer, breast cancer and leukemia, especially the Hodgkin Lymphoma (HL) followed by Non-Hodgkin lymphoma (NHL) [3]. Its rate of occurrence is 0.8 case per 100.000 pregnant women [4] with a frequency peak between 37 and 42 years old. However,this number is expected to rise because of the increasing age of women at conception, the observed increase in NHL incidence over the past two decades, and the growing incidence of HIV which has increased the risk 150 times more [3]. Studies have shown a large prevalence of aggressive forms [4], in young subjects, but an incidence abnormally high of extranodal involvement (breast, uterine and ovarian). Bone involvement is present in 5 to 15% of the cases [5]. Most frequently found while having a Large B cell lymphoma. The involvement predominates in the limbs especially the femur and the axial spine. Bone lymphoma can be primary or secondary, unifocal or multifocal. Clinically, symptoms are inconstant. The presenting symptoms of bone involvement are bone pains associated sometimes with swelling, pathological fracture, neurological signs and/ or general signs. The definite diagnosis is histological by biopsy of an accessible lesion[3]. Imaging studies are needed to stage patients with non-Hodgkin lymphoma especially cervico-thoraco-abdomino-pelvic scanner which should be carefully discussed so as that the total radiation dose delivered to the fetus be as low as possible [3].

Clinical data suggest a similar prognosis for pregnant and non pregnant women [4]. The treatment of pregnant women with aggressive non-Hodgkin lymphoma type diffuse large-cell B-cell lymphoma, although usually the same as that in non-pregnant women, needs to be modified according to gestational stage [3] and needs to be started early to avoid significant disease-related morbidity and mortality. The current standard of care is CHOP (cyclophosphamide, doxorubicin, vincristine, prednisolone) with rituximab. When diagnosed in the first trimester, the woman should be counselled to consider a medical interruption of pregnancy in view of potential teratogenicity, spontaneous abortion and fetal death [6]. Chemotherapy should be commenced. It seems reasonably safe to treat aggressive lymphoma presenting in the second or third trimester with CHOP with rituximab [7]. Administration of rituximab, an anti-CD20 monoclonal antibody, during pregnancy is not enough documented. However, its use during the second and third trimesters seems to be safe [8].

Few studies have evoked a long-term prognosis on psychomotor development and the immune hematlologic condition of infant exposure to chemotherapy in utero [9]. Concerning subsequent fertility of women, studies have brought back the frequency of a secondary sterility following a CHOP [10].

## Conclusion

Haematological cancer in pregnancy, although rare, especially non-Hodgkin lymphoma. It poses diagnostic and therapeutic challenges. Its management is complex, requires a multidisciplinary approach and should focus on survival of the mother, while minimising treatment-related fetal toxic effects.
